# Editorial Note: Heregulin-HER3-HER2 signaling promotes matrix metalloproteinase-dependent blood-brain-barrier transendothelial migration of human breast cancer cell lines

**DOI:** 10.18632/oncotarget.28900

**Published:** 2026-07-02

**Authors:** Majid Momeny, Jodi M. Saunus, Flavia Marturana, Amy E. McCart Reed, Debra Black, Gianluca Sala, Stefano Iacobelli, Jane D. Holland, Dihua Yu, Leonard Da Silva, Peter T. Simpson, Kum Kum Khanna, Georgia Chenevix-Trench, Sunil R. Lakhani

**Affiliations:** ^1^University of Queensland, UQ Center for Clinical Research, Herston, QLD, Australia; ^2^QIMR Berghofer Medical Research Institute, Herston, QLD, Australia; ^3^Mediapharma s.r.l., Chieti, Italy; ^4^Department of Cancer Research, Max Delbruck Center for Molecular Medicine, Berlin, Germany; ^5^Department of Molecular and Cellular Oncology, The University of Texas MD Anderson Cancer Center, Houston, TX, USA; ^6^Pathology Queensland, The Royal Brisbane & Women’s Hospital, Herston, QLD Australia; ^7^The University of Queensland School of Medicine, Herston, QLD Australia

The Oncotarget Scientific Integrity Office checked a data misplacement in the article: Momeny M, Saunus JM, Marturana F, McCart Reed AE, Black D, Sala G, Iacobelli S, Holland JD, Yu D, Da Silva L, Simpson PT, Khanna KK, Chenevix-Trench G, Lakhani SR. Heregulin-HER3-HER2 signaling promotes matrix metalloproteinase-dependent blood-brain-barrier transendothelial migration of human breast cancer cell lines. Oncotarget. 2015 Feb 28;6(6):3932-46. doi: 10.18632/oncotarget.2846. PMID: 25668816; PMCID: PMC4414164.

Specifically, a duplication of the α-tubulin loading control between the western blots in Figure 4C (MCF-7 cells) and Figure 7C (HBMEKs cells) was found. No additional inconsistencies were identified during our analysis.

When contacted for clarification, the corresponding author, Dr. Sunil R. Lakhani, stated that “this work was published nearly 10 years ago, and the experiments were done prior to that. We have been unable to find the original data from the experiments. Hence, we are unable to either confirm or refute the ‘irregularity’ picked up using the AI tool ImageTwin and posted to PubPeer. Since the work was done such a long time ago, we no longer have the materials to be able to repeat the experiments.”

As the identified issue cannot be corrected with original data, in accordance with the COPE guidance the journal is issuing an Editorial Note* to inform our readers and maintain the transparency and integrity of the journal’s records. The authors agree with the publication of the Editorial Note.

*COPE guidance: “Concerns raised by a complainant that do not warrant a correction or expression of concern could also be resolved by some other means (e.g., letter to the editor, or note or notification).” https://publicationethics.org/guidance/guideline/expressions-concern.

Original article: Oncotarget. 2015; 6:3932–3946. 3932-3946. https://doi.org/10.18632/oncotarget.2846

